# Results of multiple ligament reconstruction after knee dislocation——A prospective study with 95 patients and minimum 2-year follow up

**DOI:** 10.1186/s12891-021-04596-9

**Published:** 2021-10-27

**Authors:** Tao Li, Yan Xiong, Zhong Zhang, Xin Tang, Gang Chen, Qi Li, Wei Li Fu, Jian Li

**Affiliations:** 1 Department of Orthopedics, Orthopedic Research Institute, West China Hospital, Sichuan University, Sichuan Chengdu, China; 2 Department of Orthopaedic Surgery, West China Hospital, Sichuan University, No 37 Guo Xue Xiang, Chengdu, Sichuan 610041 P. R. China

**Keywords:** Knee dislocation, Multiple ligament injuries, Operative surgical procedure, Classification

## Abstract

**Background:**

There is still a lack of clinical data in arthroscopic treatment for treating multiple ligament injuries. This study aims to evaluate the clinical outcomes of patients with multiple ligament injuries undergoing treatment based on the classification stage and type of injury.

**Methods:**

A prospective, clinical trial on multiple ligament injuries was planned, which included 95 patients (58 men and 37 women; age: 42.8 ± 11.9 [range, 18–63] years) from October 2017 to June 2018. Injuries were classified into three stages (emergency stage < 24 h; acute stage: 24 h to 3 weeks, and chronic stage: > 3 weeks) and six types (KD I–VI) based on injuries time and structures, which indicated appropriate treatments. The clinical outcomes were evaluated at 2, 4, 6, 8, and 12 weeks and at 6, 9, 12 months and 24 months after surgery. The final choices in efficacy index included International Knee Documentation Committee (IKDC) score, Lysholm score, visual analog scale (VAS) score, and range of motion.

**Results:**

During the follow-up, all patients exhibited statistically significant functional improvement in the injured limb compared with their preoperative situation. The mean postoperative scores of acute stage patients at 2-year follow-up were IKDC subjective score, 77.54 ± 11.53; Lysholm score, 85.96 ± 9.39; Tegner score, 4.13 ± 1.08; and VAS score, 1.21 ± 0.76. The mean postoperative scores of chronic stage patents at 2-year follow-up were IKDC subjective score, 74.61 ± 12.38; Lysholm score, 81.71 ± 10.80; Tegner score, 3.96 ± 1.14; and VAS score, 1.71 ± 0.60. The IKDC subjective score, Lysholm score, and Tegner score were significantly improved (*P* < 0.01) and the VAS score was significantly decreased (*P <* 0.01) at 2-year follow-up. Regarding the multiple ligament injuries classification, patients with more structural damage in stages V and VI showed less progress in functional recovery than those in stages I–IV.

**Conclusions:**

This new classification with three stages and six types helps to identify the severity of injury and plan the management effectively. The outcomes were encouraging and the subjective functional results showed significant improvement at 2-year follow-up.

**Study design:**

Prospective clinical trial.

**Level of evidence:**

II

## Introduction

Multiple ligament injuries is one of the most severe situations in sports medicine. At present, the incidence rate of multiple ligament injuries is 0.02–0.2% of orthopedic injuries and < 0.5% of all joint dislocations [1]. Among the patients with multiple ligament injuries, vascular injury occurs at a rate of 7–32%, with an average of 30% [2]. In case of other injuries, it is reported that combined common fibular injury occurs in approximately 14–40% of all cases, fracture combined with knee dislocation occurs in 16% of all cases, and meniscus injury occurs as collateral damage of knee dislocation in 15–20% of all cases [2–4]. It was reported that surgical management was predictable when specifying the grade and topographic location of each ligament injury [5]. In the meantime, the failure to identify a concomitant injury can worsen the outcome of the surgical reconstruction [6]. Therefore, it is essential to use staging and individualized management of multiple ligament injuries depending on the patient’s condition.

According to the period of the injury, multiple ligament injuries were generally categorized into three phases: emergency stage, < 24 h; acute stage, 24 h to 3 weeks; and chronic stage, > 3 weeks [2–4]. Some authors suggested a subacute phase of 3–6 weeks [2–4]. The two commonly used classifications of knee dislocation were the Kennedy classification and the Schenck classification. In the Kennedy classification [7], knee dislocation was classified based on the relative displacement of tibia to the position of femur during injury. Further, injuries were classified into anterior dislocation, posterior dislocation, medial dislocation, lateral dislocation, and rotational dislocation. This dislocation classification was easy to understand and plays an important guiding role in the diagnosis and treatment of knee dislocations. However, approximately half of all knee dislocations would be reduced after the injury and therefore the initial dislocation direction of the knee joint could not be judged, rendering this classification system impractical in clinical practice. The Schenck classification system [8] classified the knee dislocations based on the assessment of the stability of the knee under anesthesia and emphasizes the relationship between the ligaments injury and knee dislocation. Two and more ligaments caused dislocation of joint, but it failed to strictly separate the ligament injury, knee instability and knee dislocation. Meanwhile, patients with different surgical procedures have combined injuries, and complete evaluation and analysis is lacking, which cannot fully guide the clinical treatment.

Thus, domestically and internationally, there is an ongoing debate about multiple ligament injuries concerning the analysis of stage, appropriate treatments during different stages, and optimal postoperative recovery protocol [9]. Consequently, settling this debate by establishing universal criteria acquired by high-quality research with a large number of participants is of crucial importance. Nevertheless, obtaining an adequate number of applicable participants is difficult, because multiple ligament injuries has a relatively low rate of incidence to encounter enough patients to study and it is difficult for research centers to perform a comprehensive research with an adequate case pool. Since 2015, our team has led a study on multiple ligament injuries management, wherein about 400 patients were treated according to different stages of multiple ligament injuries. Therefore, a full new set of diagnostic, treatment, and recovery protocols on multiple ligament injuries which led a significant functional improvement was summarized. From 2017 to 2018, a prospective study had initiated and hereby all multiple ligament injuries patients were provided with those new classification stage and type protocols. This study aimed to provide instructive clinical evidence and corresponding optimal treatments with designated classification stage and type of injury for multiple ligament injuries.

## Methods

### Description of the classification system

In this study, the injury period of multiple ligament injuries were classified into three stages: emergency stage (<24 h), acute stage (24 h to 3 weeks), and chronic stage (> 3 weeks). As for the injury classification, multiple ligament injuries were categorized by the location of injury, cause of injury, and whether a fracture occurred. Compared with the traditional method of classifying patients into those having medial and lateral dislocation by the direction of the brute force applied, collateral damage from the injuries were placed greater consideration (Table 1). The differences between KD- V and KD-VI were bony fracture forms in multiple ligament injuries. In KD- V type, bony fracture block connected to at least one major ligament, such as anterior cruciate ligament (ACL), posterior cruciate ligament (PCL), medial collateral ligament (MCL), or lateral collateral ligament (LCL). Thus, bony fracture block affected the joint support and stability, which led to knee dislocation. Meanwhile, in KD-VI type, multiple ligamentous injuries caused to knee dislocation, combined bony fracture around knee. no major ligaments (ACL, PCL, MCL, LCL) attached on bony fracture block, which did not contribute much to the stability of knee. Therefore, the relationship of knee dislocation, bony fracture and multiple ligament injuries in KD- V and KD-VI types were very important for knee restoration. A more detailed category was shown in Table 1.

**Table 1:** The classification of the knee dislocation

Stage	Emergency (< 24 h) Acute (24 h to 3 weeks) Chronic (> 3 weeks)			
**Type**	**Subtype**		**Structural damage**	**Other structural damage**
KD-I	Anterior		ACL + PLC + PMC	Open wound
	posterior		PCL + PLC + PMC	Vascular injury
KD-II	Medial		PMC + ACL + PCL	Nerve injury Meniscus injury
	Lateral		PLC + ACL + PCL	
KD-III	MC	Anterior	PMC + ACL + PCL	Cartilage injury
		Posterior		Knee joint reduction
	LC	Anterior	PLC + ACL + PCL	Fractures in other parts
		Posterior		
KD-IV	Simple		ACL + PCL + PMC + PLC	
	Complex		ACL + PCL + PMC + PLC +gastrocnemius muscle avulsion +patellar tendon/patellar fracture	
KD-V	Femur		Fracture dislocation	
	Tibia			
KD-VI	Femur		Dislocation with fracture	
	Tibia			

*KD* knee dislocation, *ACL* anterior cruciate ligament, *PCL* posterior cruciate ligament, *PMC* posteromedial complex, *PLC* posterolateral complex, *MC* medial corner, *LC* lateral corner

### Basic patient information

The prospective research was registered at http://www.chictr.org.cn/index.aspx (ChiCTR-OIC-16008306 19/04/2016) and approved by the clinical research ethics committees (Sichuan University ID:2016–99 11/1/2016). All patients or their legal representatives signed the informed consent. All methods were carried out in accordance with relevant guidelines and regulations and informed consent was obtained from all subjects or, if subjects are under 18, from a parent and/or legal guardian. Criteria for including, excluding patients and terms of termination were the following.

Including criteria:

1. Patients with knee dislocation
2. Three or more major ligaments injured in knee joint shown by MRI
3. Knee joint demonstrates obvious anterior/posterior and lateral/medial instability by physical examination
4. No other severe physical injury that hinders limb functions
5. Patients voluntarily agree to treatment protocol and are willing to cooperate in rehabilitation and follow-up examination

Excluding criteria:

1. Patients with severe osteoarthritis
2. Patients with combined complications which significantly hinders limb functions
3. Patients that can’t be treated or can’t cooperate

Terms of termination:

1. Patients don’t cooperate and demand quitting
2. Patients suffer from second other function-hindering injuries that require a second surgery
3. Patients decease due to other conditions
4. Patients evaluated by professionals and ruled no longer fit in our research such as unable to complete the follow-up on time.

**Fig. 1:**
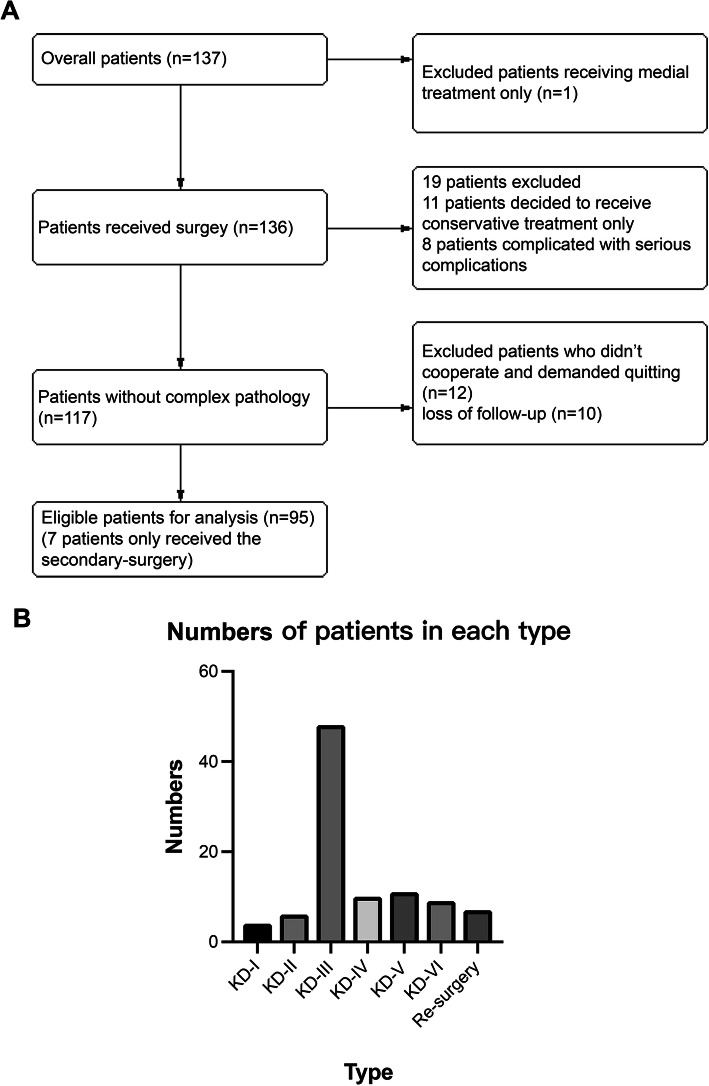
**(A-B)**: Patient flow through the study (**A**). Number of patients with each dislocation type (**B**)

137 patients with multiple ligament injuries who were treated at our hospital were enrolled from October 2017 to June 2018. Of those, 95 patients (58 men, 37 women) completed the trial (12 patients decided not to receive the surgery and 8 patients complicated with serious complications). The average age of patients was 42.8 ± 11.9 years (range, 18–63 years). Of the 95 patients, 46 had left knee dislocation, 42 had right knee dislocation, and 7 patients had both knees dislocated. On classifying the patients based on the time period, there were 7 patients in emergency stage (< 24 h), 54 patients in acute stage (24 h to 3 weeks), and 27 patients in chronic stage (> 3 weeks),7 patients only received the secondary-surgery. Of the 95 patients, 48 were classified as KD-III type. 15 patients had a diagnosis of nerve injury, 10 had collateral vascular injury, and 20 patients had fracture. Detailed information is provided in Fig. 1**.**

### Preoperative examination

All patients were examined using X-ray and MRI to obtain accurate evaluation concerning whether the joint was aligned well, a fracture had occurred or collateral tissue damage, and so on. In addition, patients with suspected embolism or vascular damage (10 of the 95 included patients) were submitted to angiography to identify the specific location of the clot. Nerve injuries, especially common fibular nerve injury, were also assessed.

### Operative methods

1. Treatment Strategy for Different Stages

**Fig. 2:**
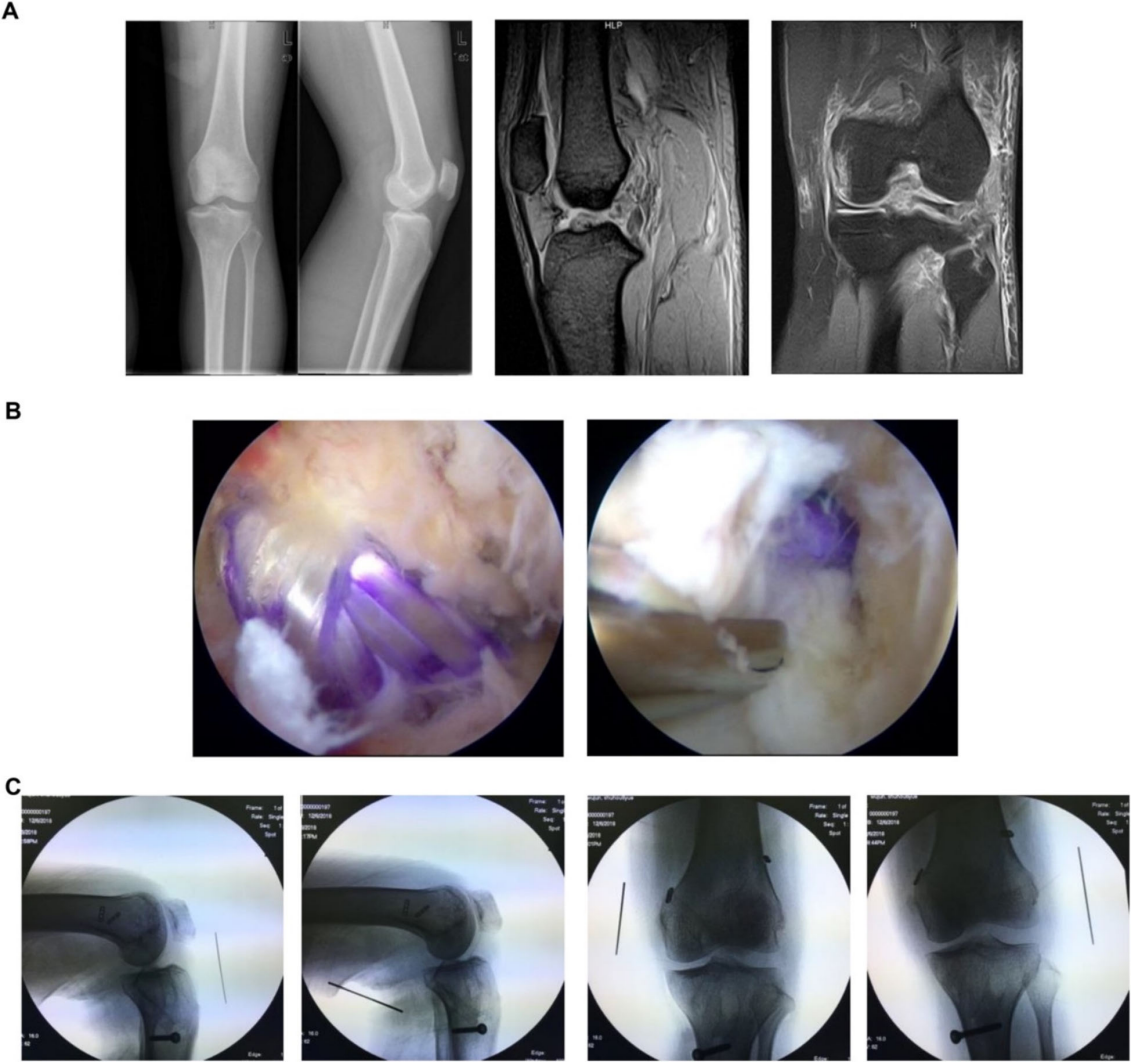
**(A-C)**: A 48-year-old male patient suffered movement obstruction for 18 days after being hit by a heavy object on the superior part of the left knee. The whole diagnosis was acute stage KD-III: ACL + PCL + LCL, no fracture or cartilage injury, and accompanied by medial and lateral meniscus injury (**A**). The surgery was performed including arthroscope probing in left knee + ACL/PCL reconstruction + LCL repairing + medial and lateral meniscus repairing (**B**). An X-ray stress examination showed that the knee maintained good stability at the last follow-up (**C**). Functional scores after 1 year were International Knee Documentation Committee score: 75; Lysholm score: 83; and Tegner score: 4

**Fig. 3:**
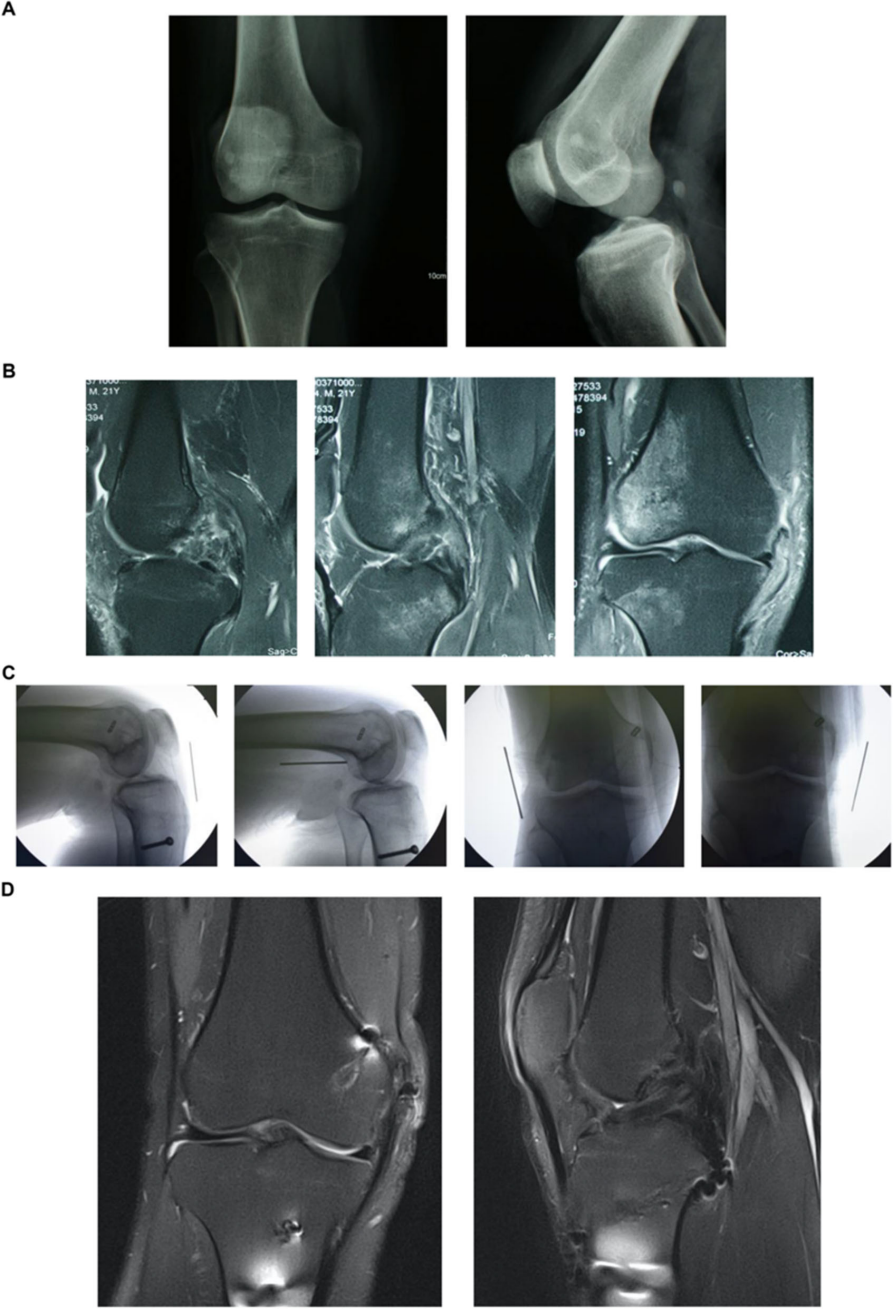
**(A-D):** A 21-year-old male patient suffered painful swelling of the right knee with functional obstruction for more than 2 months. Clinical diagnosis was chronic stage complicated right knee dislocation (KD-III: ACL + PCL + MCL) with bone contusion, no fracture or cartilage injury, and accompanied by medial and lateral meniscus injury (**A, B**). The surgery performed included arthroscopic probing in the right knee + ACL/PCL reconstruction + MCL repairing + medial and lateral meniscus repair. An X-ray stress examination showed that the knee maintained good stability at the last follow-up. The appearance of the ligament repair (ACL, PCL) and reconstructions (MCL) were as expected at 1 year after surgery (**C, D**). Functional scores after 1 year were International Knee Documentation Committee score: 73; Lysholm score: 85; and Tegner score: 5

For emergency stage patients (< 24 h), an immediate and comprehensive assessment of the patient’s vitals would take precedence over the management of the affected limb if there was a life-threatening injury. The focus would be on keeping the knee realigned while immediately dealing with complications such as open injury, vascular damage, and osteofascial compartment syndrome. In the meantime, the vascular status as well as other additional injuries can influence the timing of surgery [10, 11]. In case of an open wound, debridement would be performed as soon as possible, strictly according to the principle of orthopedic open wound management. When the soft tissue is not in good condition, it is often accompanied by severe swelling and ecchymosis, and it may have a high risk of infection, so delayed surgery may be a better option to recover soft tissue. If necessary, surgeons would manage to prevent potential wound healing intraoperatively, such as practicing meticulous skin and soft tissue management with full tissue skin flaps. For acute stage patients (24 h to 3 weeks), the focus would be on repair and reconstruction, if necessary, with the aim to sustain joint realignment and achieve long-term joint stability **(**Fig. 2**)**. Chronic stage patients (> 3 weeks) with multiple ligament injuries were difficult to operate on; therefore, the primary concern would be correction of the alignment and restoration of knee flexibility by focusing on restoration of the stable structure of the knee joint motor central axis **(**Fig. 3**)**. There are controversies in the timing of surgery. Some argued that delayed ligament reconstruction approach is better while some reported that acute primary repair of extraarticular ligaments may reduce the need for subsequent cruciate ligament reconstruction [12, 13]. Since there is no general consensus, we chose to stick to our protocol.

2) Order of Ligaments Reconstruction

**Fig. 4:**
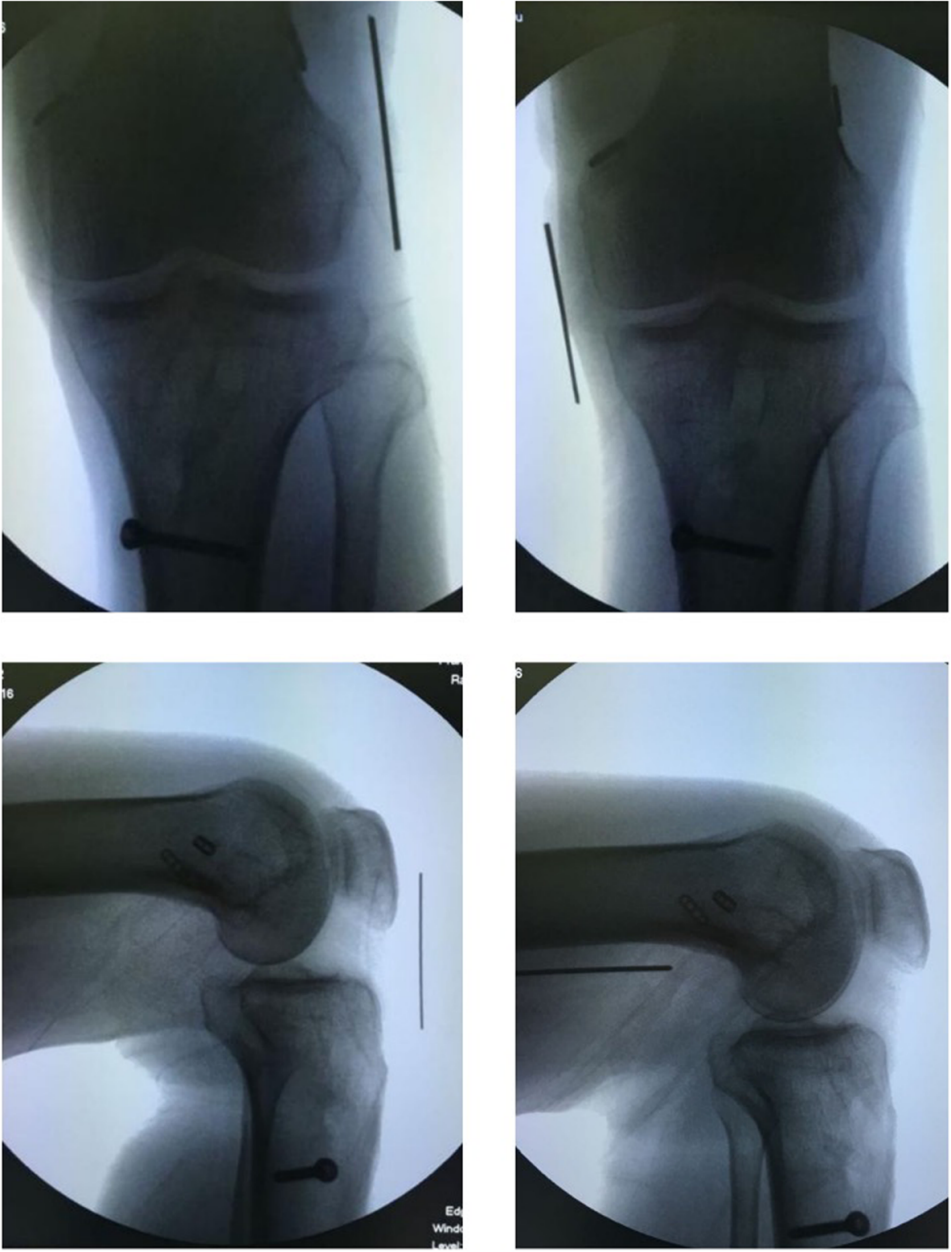
The postoperative X-rays of a 45-year-old man with PMC injury

**Fig. 5:**
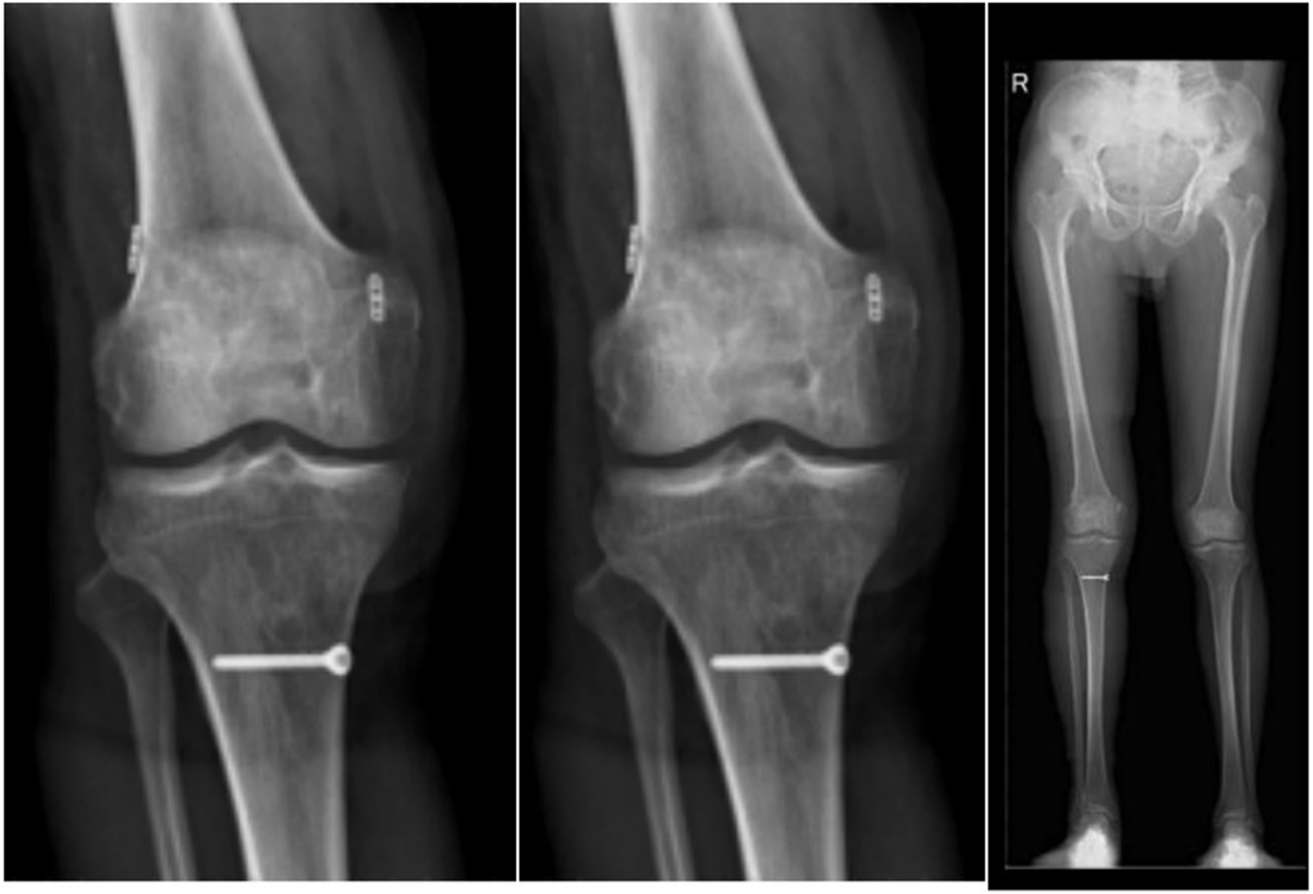
The postoperative X-rays of a 59-year-old man with PLC injury

For patients with multiple ligament injuries, the order in which the various damaged ligaments w reconstructed is pivotal to treatment efficacy. Currently, the optimal order is ambiguous. In this study, we have a similar order with the LaPrade et al. [14],the first reconstruct was PCL in the case of ACL/PCL/medial and lateral complex injures. Secondly, the reconstruction of the posterolateral structure among the remaining structures was prioritized because of maintaining varus stability of the knee joint. The next structure was ACL. Reconstruction of the MCL would be considered when the ligament parenchyma is damaged, not when femoral condyle “peel off” occurs. The lateral structure was prioritized over the other structures when considering fixation, and then PCL, medial structure, and last was ACL **(**Figs. 2, 3, 4, and 5, Table 2).

**Table 2:** The Order of Ligaments Reconstruction

Operation sequence of ligament repair and reconstruction	
1. Arthroscopic exploration and treatment of meniscus and articular cartilage injury.	
2. Establishing tibial tunnel of posterior cruciate ligament.	
3. Establishing femoral tunnel of posterior cruciate ligament.	
4. Establishing Femoral tunnel of anterior cruciate ligament.	
5. Establishing Tibial tunnel of anterior cruciate ligament.	
6. Tighten the posterior cruciate ligament graft at full extension and fix it at 90 degrees flexion.	
7. Tightened and fix anterior cruciate ligament graft at full extension.	
8. Repair and enhance the deep and shallow medial collateral ligament in 30 degrees flexion with mild external rotation. Then tighten and fix the medial collateral ligament.	
9. Tighten and fix posterior oblique ligament and posterior medial joint capsule near complete extension	
10. Repair, enhancement or reconstruction of PLC in 30 degrees of knee flexion, with valgus and mild internal rotation.	
a. Order of ligament tightening and fixation: PCL—ACL—PLC—PMC b. Fracture reduction and fixation is prior to ligament reconstruction and repair.

3) Surgical techniques for reconstruction

All avulsed structures were addressed by the autografts. The single-bundle technique was used for both PCL and ACL reconstructions, but the double-bundle technique was also a choice if necessary. Only 2 of the included patients received artificial-ligament implantations The PCL- and ACL- drill tunnels were placed in a standard fashion. For the posterolateral corner, there are two key structures, which are lateral collateral ligament (LCL) and the popliteofibular ligament. Which reconstruction technique we applied depends on the patients’ situation, but in most cases, the anatomical reconstruction was adopted. The LCL was reconstructed by transposition of the intermediate one-third of the biceps tendon and fixation to the normal lateral epicondylar insertion of LCL. When reconstructing the popliteofibular ligament, the tibial allogeneic tendon was tensioned through the tibial and fibular tunnel and fixed at the equal-length point of the lateral condyle of the femur using screws and spiked-washer nails. The fibular tunnel runs from the anterolateral to the posterolateral. The reconstruction of MCL was performed simultaneously with that of POL. Bone-penetrating suture technique was applied for starting and ending point injury. If there were still medial collateral ligament injuries, they were directly repaired through a medial curvilinear incision. In this study, we had a similar surgical management for multiple ligament injuries with Harner CD et al. [15].

### Postoperative rehabilitation

Immediately after operation, the patients managed limb swelling and began isometric strengthening training. If the PCL, MCL, or LCL were repaired, a knee brace for external fixation with 0° extension was recommended to wear for 4–6 weeks before weight-bearing training. It was reported that postoperative protection of multiple knee ligament reconstructions in a hinged external fixation device may lead to more favorable static stability than postoperative brace immobilization [16], so at 6 weeks, patients were permitted to start partial weight-bearing activities as well as close chain range of motion with braces and flex the knee to 90°. Nevertheless, the hinged brace was applied after the surgery if necessary. Notably, for patients with posterolateral complex injury and posterior structural injury, weight-bearing training for the injured knee cannot be arranged early. For such patients, weight-bearing walking would start 8 weeks after surgery. At 3 months, presuming good healing of the soft tissue structure suture repair, patients were cleared for limited exercise, such as housework and walking, after the knee was able to flex > 120°. In addition, specific muscle training according to the different recovery stages would be performed. Ninety of 95 patients (94.7%) were satisfied with their mobility after 6 months of recovery and were expected to participate in ordinary sports after 9–12 months.

### Evaluation of therapeutic effectiveness

All patients were reexamined at 2, 4, 6, 8, and 12 weeks and 6, 9, 12 months and 24 months after surgery. They were also scored at 3, 6, 9, 12 months and 24 months after surgery. Knee flexibility, stability, and functional scoring and imaging methods (X-ray and MRI at 6, 12 months and 24 months to obtain accurate evaluation concerning whether the joint was aligned well, a fracture had recovered or collateral tissue situation, and so on) were performed. A follow-up survey was conducted by two authors (T L and Y X) in the Outpatient Department. International Knee Documentation Committee (IKDC) grade, IKDC subjective score, Tegner score, and Lysholm score were observed to make quantified comparisons between preoperative and postoperative knee function. Postoperative scoring was conducted by two authors (T L and Y X) in the Outpatient Department during follow-up.

The prospective trial was registered at http://www.chictr.org.cn/index.aspx (ChiCTR-OIC-16008306) and approved by the clinical research ethics committees (Sichuan University ID:2016–99).

### Statistical analyses

Statistical analyses were undertaken by SPSS 22.0 software (SPSS Inc., Chicago, IL, USA). Continuous data are presented as a mean ± standard deviation (SD). ShapiroeWilk test was used to prove whether the data belong to normal distribution, and (W-H) rank sum test was used if the data do not conform to normal distribution. For data conforming to normal distribution, Leven test was used to evaluate the homogeneity of variance. The 95% confidence interval (CI) to describe the percent limit of data. Normality data, Changes in preoperative and postoperative IKDC score, Lysholm score, visual analog scale [VAS] score, and Tegner score were analyzed using a paired samples t test. A *p* value less than 0.05 was considered significant, and a *p* value less than 0.01 was considered highly significant for all statistical tests.

## Results

### General results

Ninety-five patients were included in the study, with an average operative time of 1.5–4 h. The enrolled patients exhibited good wound healing without infection. In the study, three acute stage patients and four chronic stage patients received ligament revision surgery (7/95, 7,3%), including two PCL, three PLC, and two ACL. Three acute stage patients and one chronic stage patient accepted knee manipulation under anesthesia at 2–3 months after surgery.

### Functional scoring results

**Fig. 6:**
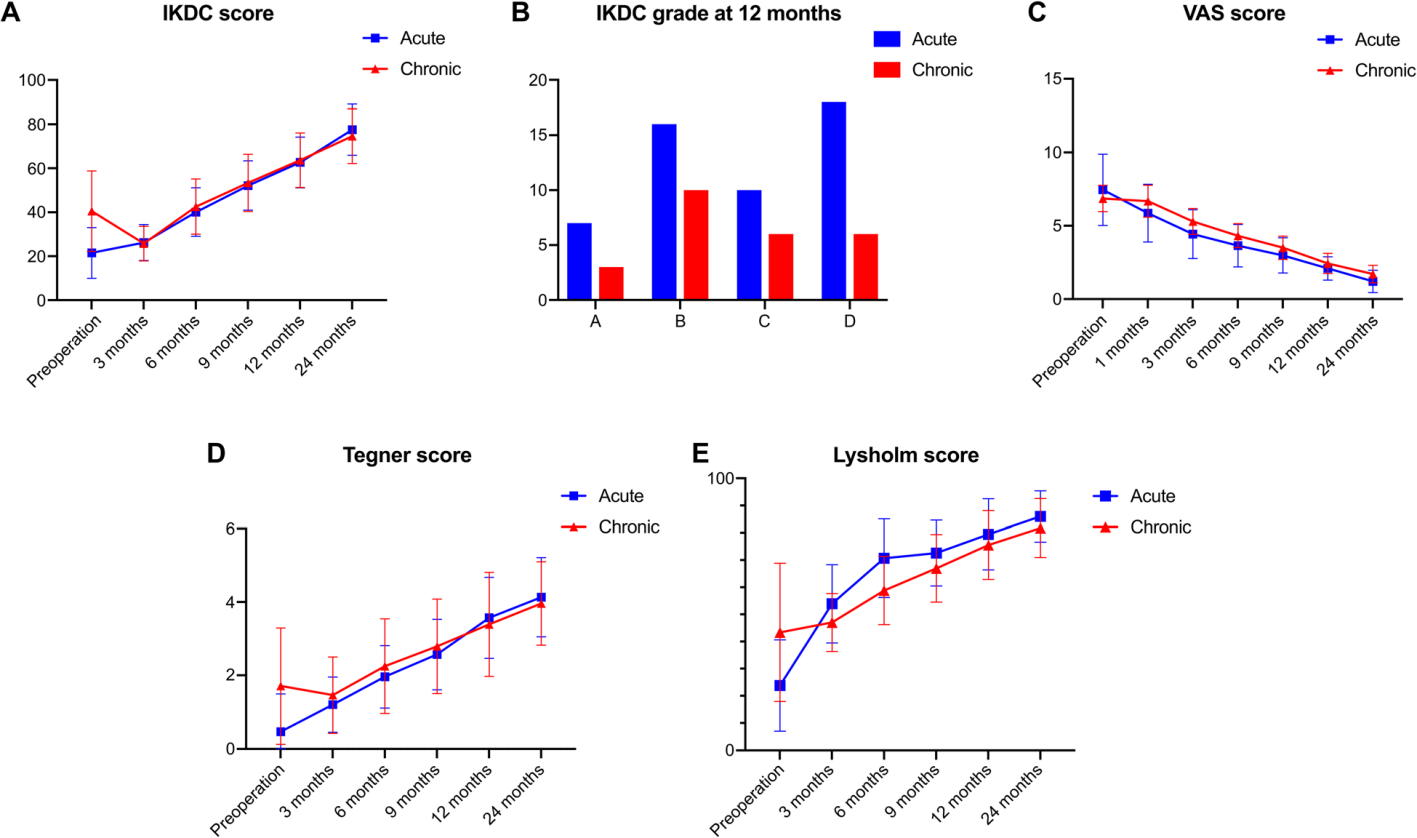
**(A-E)**: The 2-year patient-reported outcomes for the acute stage and chronic stage groups over time: International Knee Documentation Committee (IKDC) score (**A**), IKDC grade at 12 months (**B**), visual analog scale (VAS) score (**C**), Tegner score (**D**), and Lysholm score (**E**)

All patients exhibited significant improvement in functional scoring 1 year after surgery, indicating satisfactory effects of the surgery and postoperative recovery of knee mobility. For patients in the different classification stages, postoperative knee function was significantly improved. The mean postoperative scores at 2-year follow-up were as follows: The mean postoperative scores of acute stage patients at 2-year follow-up were IKDC subjective score, 77.5 ± 11.5; Lysholm score, 86.0 ± 9.4; Tegner score, 4.1 ± 1.1; and VAS score, 1.2 ± 0.8. The mean postoperative scores of chronic stage patents at 2-year follow-up were IKDC subjective score, 74.6 ± 12.4; Lysholm score, 81.7 ± 10.8; Tegner score, 4.0 ± 1.1; and VAS score, 1.7 ± 0.6. The IKDC subjective score, Lysholm score, and Tegner score significantly improved (*P* < 0.01) and the VAS score significantly decreased (*P <* 0.01) at 2-year follow-up compared with the preoperative values. Details are listed in Fig. 6 (A-E).

### Outpatient department follow-up results

**Fig. 7:**
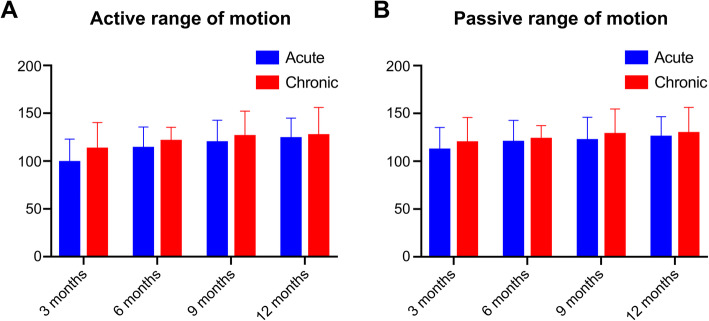
(**A**-**B**): The 2-year patient-reported outcomes for the acute stage and chronic stage groups over time: Active range of motion at 12 months (**A**) and Passive range of motionat 12 months (**B**)

Ninety-three of 95 patients (97.9%) reached Level V in the muscle strength at 1 year. The major passive range of motion (PROM) of the patients was restored to normal. Five patients had a Level I positive anterior drawer test (ADT) and two patients had a Level I positive posterior drawer test (PDT). Four patients had a positive 0° genu valgus test and one patient had a positive 0° genu varus test. The details about the active ROM and PROM are provided in Fig. 7(A-B).

### Other results

Patients with embolism were treated with strict postoperative anticoagulant therapy and had no recurrence of the severe complication. 15 patients with common fibular nerve injury were treated with decompression and postoperative exercise and showed no signs of any complications. Meniscus injuries were sutured and all patients healed completely. With the 20 patients with fracture, the reduction and fixation of fracture was done.

## Discussion

The most important findings of the present study were superior treatment efficacy can be achieved by categorizing the injury based on the stage and type and applying the corresponding optimal treatment. The incidence of multiple ligament injuries is relatively low, but the damage is serious and its treatment is difficult. Furthermore, it may have serious consequences on the limb function of the patient [2–4]. According to epidemiological studies, knee dislocation accounts for 0.8% of all orthopedic injuries [2–4]. The stage and type of injury are critical for the diagnosis and treatment of multiple ligament injuries. Currently, the Schenck and Kennedy classification systems are the two major systems for classification of knee dislocation; the Schenck system focuses on the multiple ligament injuries and the Kennedy system focuses on the direction of the dislocation. In the previous study, the outcomes showed that complications and fractures played an important role in the diagnosis and treatment of knee dislocation and affected the clinical results [2–4, 17–19]. Therefore, in this study classification of multiple ligament injuries added complications and fractures factors.

However, early repairing versus delayed reconstruction remains controversy regarding repair or reconstruction of ligament rupture. In the prior literature, Mariani et al. [20] and Fresch et al. [21] found no difference in clinical results when suture repair and reconstruction were compared. Levy [22] reported that early operative treatment of the multiple ligament injur yields improved functional and clinical outcomes compared with the delayed surgery. Hohmann [23] concluded that patients with acute stage (mean 10.6 days) reported superior clinical outcomes compared with those with chronic stage. And a recent systematic meta-analysis [24] suggested that the functional outcome score showed better results in the early surgical treatment than in the delayed group, with a higher mean Lysholm scores (89 vs 82) as well as a higher percentage of excellent IKDC scores (57 vs 41). However it was clear that operative repair or reconstruction showed superior clinical and functional results when compared with conservative treatment [25, 26]. In this study, ligament injury in the mesenchymal parts of the knee should be treated with reconstruction. Injuries to the LCL and MCL attachments should be treated with suturing, which demonstrated better clinical outcome. In the clinical practice of multiple ligament injuries treatment, when the ACL and PCL are injured simultaneously, if only one ligament can be reconstructed or is prioritized to be reconstructed first under certain circumstances, PCL should be prioritized. It is highly important for stabilizing the central axis of the knee and even the line of gravity of the lower limb [27, 28]. As for the other three ligaments, LCL was repaired prioritizing. In general, multiple studies have indicated that at least three out of the four ligaments need to be intact to maintain stability when standing [14, 29, 30].

After 2-year of short-term follow-up, patients showed favorable clinical results. To the best of our knowledge, studies on multiple ligament injuries are based on sample sizes of approximately 8–85 and follow-up periods of 1–13 years [31–37]. The reconstruction studies reported similar clinical results; Lysholm scores ranged from 75 to 90 [32–34, 36–39], Tegner scores ranged from 4 to 6 [33, 34, 36–39], and IKDC scores ranged from 60 to 85 [34, 36–38]. In this study, an advanced comprehensive multiple ligament diagnostic and treatment system was performed, meanwhile postoperative rehabilitation was more targeted and effective. Thus outcomes were satisfactory at 2-year follow-up.

In the literature, the postoperative infection rates were reported as 0.5–0.9% [35, 40]. In this study, patients did not develop infections because of timely wound management in the emergency and preoperative stages. Two patients had knee decompression surgery twice, but no patients experienced any infection. Another reason for a second surgery was knee was rigid after surgery and needs to be manipulated under anesthesia [35, 40]. Other reasons for a second surgery were stiffness and transplant failure [38, 41]. Therefore, an appropriate rehabilitation program designed by a surgeon and a therapist could effectively help in avoiding these complications. During the early rehabilitation procedure, the patients are required to use braces but not hinged external fixator. The hinged external fixator, as an invasive fixation device, may lead to serious aesthetic and psychological problems. Furthermore, infection is the most frequent complication because of the pin insertion site, which occurred in up to 40% of patients when used the hinged external fixators. Patients with poor soft tissue are not suitable for the hinged external fixator. It is reported that the use of rigid bracing immobilization showed remarkable clinical results in some researches [42–44]. Although Angelini [45] and Stannard [46] reported that the use of hinged external fixator showed good clinical outcomes, they had small samples and followed a more aggressive rehabilitation protocol. But we intended to use a more moderate rehabilitation protocol based on the tissue healing timeline, which may reduce the abnormal stress for reconstructed structures [38, 41].

However, this study has some limitations. First, this study had only 1–2 years of follow-up. Therefore, the outcomes after a longer period of follow-up are needed. Second, because of the diversity and complexity of the injuries, this study did not establish a control group. In addition, because of the low number of cases reported by other co-research centers, this was a single-center study of 95 patients. More cases from other centers will be included in the next study. In our study we found that for the emergency stage patients, the main purpose is to save lives, restore limb blood supply, save limbs, close open wounds, and maintain joint realignment. Treatment of acute stage patients should be based on the diagnosis classification; repair and reconstruction of the knee ligament should stabilize the structure and restore joint function. This is the most important period for functional joint recovery. For patients in the chronic stage, the treatment difficulty is relatively high. It is necessary to restore joint stability as much as possible, repair and reconstruct to stabilize the structure, and improve joint function.

## Conclusion

This new classification can better identify the severity of the injury to plan the management better. Besides, the patients in acute stage showed greater clinical outcomes than these in chronic stages.

## Data Availability

The datasets used and/or analyzed during the current study are available from the corresponding author on reasonable request.
